# Cross-Linked Polyimide Aerogels with Excellent Thermal and Mechanical Properties

**DOI:** 10.3390/gels10100667

**Published:** 2024-10-19

**Authors:** Haoran Qian, Zhiqi Li, Song He

**Affiliations:** 1 Mining Engineering Department, Shanxi Institute of Technology, Yangquan 045000, China; qianhaoran@sxit.edu.cn; 2 School of Safety Science and Emergency Management, Wuhan University of Technology, Wuhan 430070, China; 330667@whut.edu.cn

**Keywords:** polyimide aerogel, cross-linking, freeze drying, three-dimensional construction, thermal insulation properties

## Abstract

With the increasing development of productivity, new materials that allow for the efficient use of energy are slowly becoming a sought-after goal, as well as a challenge that is currently being faced. For this reason, we have made aerogels as the target of our research and prepared different series (CLPI (1–5)) of cross-linked polyimide aerogels by mixing and cross-linking the heat-insulating cross-linking agent 1,3,5-tris(4-aminobenzylamino)benzene (TAB) with polyamic acid solution. We created a three-dimensional spatial organization by using vacuum freeze-drying and programmed high-temperature drying, then controlled the concentration of the polyamidate solution to investigate the concentration and TAB’s influence on aerogel-related properties. Among them, the shrinkage is reduced from 40% in CLPI-1 to 28% in CLPI-5, and it also shows excellent mechanical characteristics, the highest compression strength (CLPI-5) reaches 0.81 MPa and specific modulus reaches 41.95 KN m/Kg. In addition, adding TAB improves the aerogel thermal resistance, T_5_ in N_2_ from PI-2 519 °C to CLPI-2 556 °C. The three-dimensional network-type structure of the aerogel shows an excellent thermal insulation effect, where the thermal conductivity can be as low as 24.4 mWm^−1^ K^−1^. Compared with some protective materials, cross-linked polyimide aerogel presents better flame-retardant properties, greatly improving the scope of its application in the industrial protection.

## 1. Introduction

From what we can see, the global demand for power sources is increasing, and the supply needs to be efficient to meet the needs of people, and how to use the energy more efficiently is an urgent need to deal with [1,2,3,4,5,6]. New heat insulation materials can effectively solve this problem. Aerogel has emerged as an ideal thermal insulation material in recent years [7,8,9,10,11]. Most of them are obtained by sol–gel method, removing the solvent within the pores and replacing it with air. As a result, the silica aerogels possess relatively rich porosity, high specific surface area, and low thermal insulation (~20 mWm^−1^ K^−1^) [12,13,14,15,16]; however, poor mechanical properties limit their application.

However, relative to inorganic aerogels, organic aerogels such as polyimide aerogels [17,18] and cellulose-based aerogels [19,20,21] show excellent mechanical properties. Polyimide, as an applied material with excellent mechanical properties, such as being flame retardant and its heat-insulating effects, is widely used in in the fields of aerospace, protective apparel, electronic sensing, and automotive batteries [22,23,24].

The sol–gel method is usually applied for polyimide aerogel preparation where the organic solvents need to be super-critically extracted with liquid CO_2_ to form the pore structure [25,26], but it is complicated and expensive in operation. In addition, it will also cause pollution to the environment. On the other hand, a freeze-drying method is also used for polyimide aerogel preparation, which is simpler. The preparation process involves ice sublimation from the frozen solvent under vacuum. Different pore structures can be obtained by controlling the direction of growth of the ice crystals during freezing [27]. The samples can still suffer from volume shrinkage and reduced thermal stability because of the formation of ice crystals and chain-to-chain interactions during thermal imidization reaction. Therefore, some researchers introduced cross-linking agents in the aerogel precursor solution to effectively improve thermal stability performance. For example, Liu et al. [28] filled aramid fibers, and the rapid decomposition temperature of the samples increased from 435 °C to 457 °C; however, the inorganic filler was added as a solid heat transfer medium, and it did not significantly change the heat transfer effect, and the thermal conductivity of PI-3 is 34.1 mWm^−1^ K^−1^. Fan et al. [29] filled modified silica nanoparticles as a cross-linking agent into the precursor, and the thermal insulation performance and thermal decomposition temperature are greatly improved, but the brittle silica nanoparticles greatly diminished the compression resilience of aerogels. Yin et al. [30] assembled chitosan-black scale (CS-BP) coating layers in polyurethane foam (PUF), and the fire resistance was improved, and the mechanical properties were not destroyed. However, the initial decomposition temperature reached up to 280 °C, limiting the range of use at high temperature. Yuan et al. [31] prepared horizontally oriented BT@PDA/PI nanocomposite films using an in situ polymerization process. It exhibited excellent temperature stability (T_5_% = 546 °C), but also had a certain degree of brittleness.

Instead, we performed cross-linking by adding a cross-linker, 1,3,5-triaminobenzoxybenzene (TAB) [32], to the network structure; this gives the aerogels better thermal performances while ensuring its excellent mechanical characteristics. Consequently, we made a preparation for water-soluble polyimide salts with dianhydride, diamine, cross-linking agent, and organic solvents. Next, we prepared polyimide aerogels containing different amounts of polyamidates by dissolving them in alkaline solution with triethylamine, freeze-drying and programmed heating them, to investigate the relevant properties of the aerogels as well as the effects of different concentrations on the properties.

## 2. Results and Discussion

**Figure 1:**
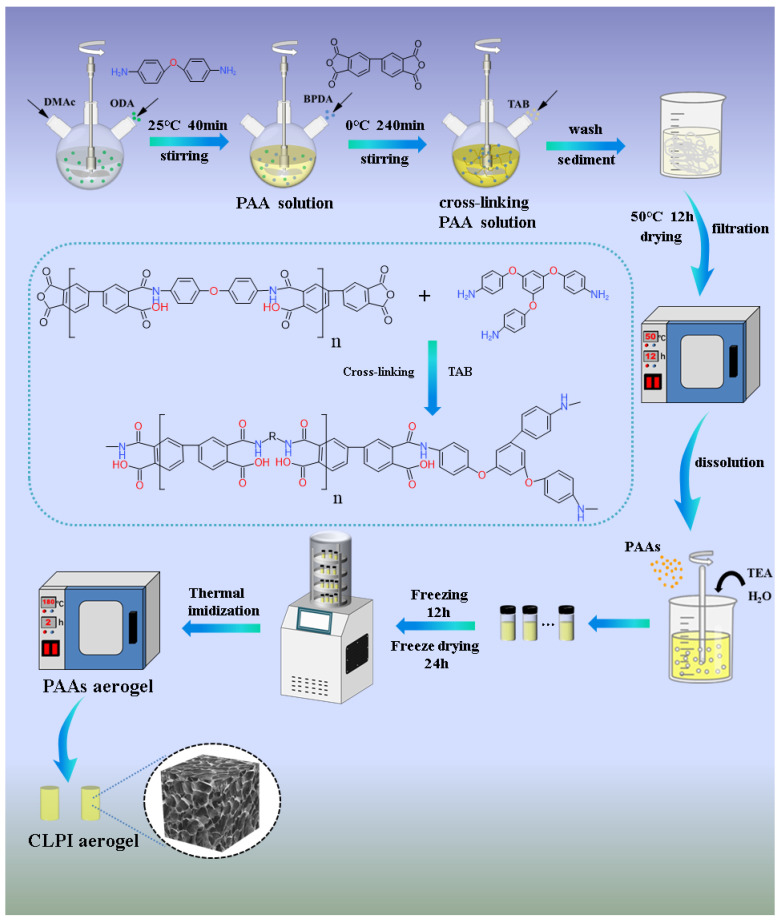
Preparation of cross-linked polyimide aerogel.

**Figure 2:**
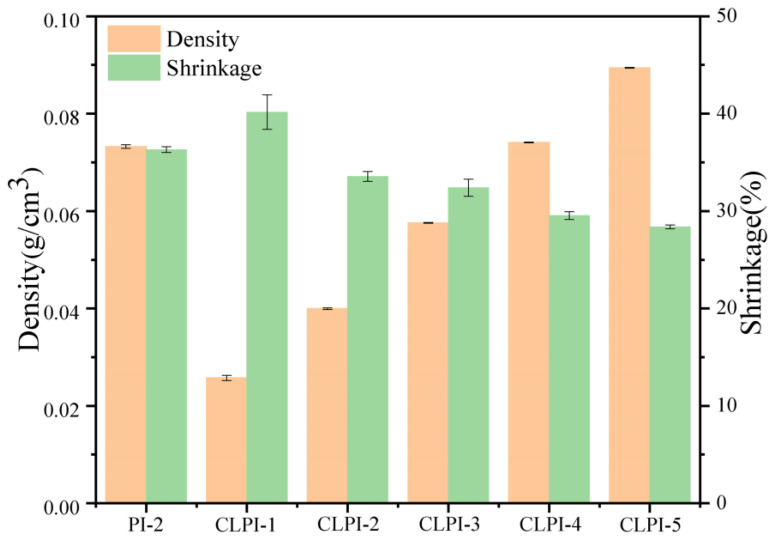
Densities and shrinkages of PI-2, CLPI1-5.

**Figure 3:**
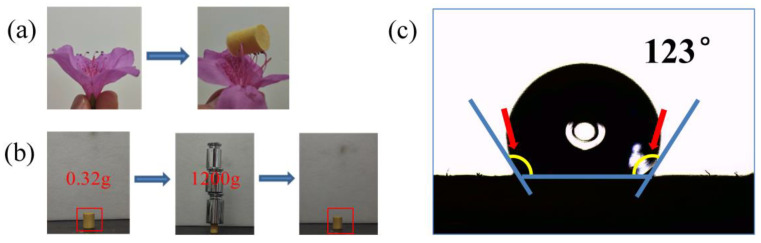
(**a**) Aerogel photographs of stamens; (**b**) before and after photographs of the aerogel bearing weight; (**c**) contact angle of water on aerogel surface.

Figure 1 represents the whole process of preparing CLPI aerogels, which were prepared by alkaline solubilization of polyamide PAAs prepared from dianhydride (ODA) and diamine (BPDA) in aqueous TEA solution. Finally, we obtained the PI aerogels using a low-cost, environmentally friendly, and multi-batch freeze-drying method; during the stirring of the polyamic acid solution, a cross-linking agent TAB was added to cross-link with the polyamic acid solution. The amino group of TAB combines with the anhydride group at both ends of PAA to obtain a three-dimensional network-type structure [33], realizing distinguished thermal insulation performances and heatproof stabilities of CLPI aerogels. Using deionized water as precipitant, displacing agent, and aging agent, the polyamic acid solution is completely converted to filamentous precipitation, and the residual organic solution is washed away, making the structure denser and more stable. The cost is greatly saved. In Figure 2, the gradual increase in density of CLPI1-5 is accompanied by a gradual decrease in shrinkage. The density of CLPI-1 reduces to 25.72 × 10^−3^ g/cm^3^, 28% of CLPI-5. The addition of TAB effectively reduces the shrinkage and density of PI, possibly because it stabilizes the overall structure while also ensuring its quality. As shown in Figure 3a,b, the aerogel can be stabilized at the top of the stamen. A 0.32 g sample of aerogel can hold nearly 3700 times the weight and cannot collapse, indicating that the aerogels are lightweight and pressure resistant. In addition, in Figure 3c, the hydrophobic angle of this kind of material surface was measured to be about 120°, indicating that the aerogel possesses favorable hydrophobicity.

**Figure 4:**
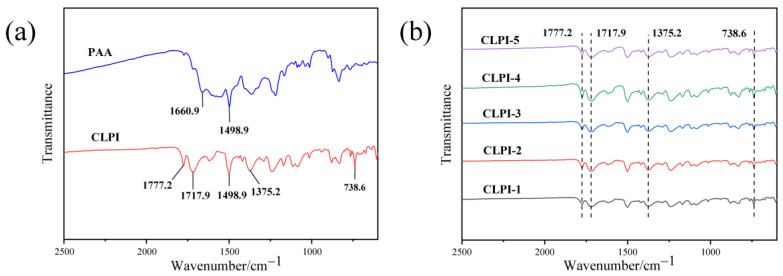
(**a**) PAA aerogel and PI aerogel FT-IR; (**b**) 1–5% concentrations of CLPI aerogels FT-IR.

To thoroughly explore the chemical structure of CLPI aerogels, we used FT-IR spectroscopy to analyze the structure of aerogel precursors and aerogels with different contents. As illustrated in Figure 4, the CLPI precursor was contrasted. From Figure 4a, there is an obvious characteristic peak near 1498.9 cm^−1^, and it is the characteristic peak of benzene ring; this characteristic peak has not disappeared or weakened before and after high temperature heating and drying, it still exists stably. We observe a characteristic peak closing to 1660.9 cm^−1^ for PAA aerogel, which is attributed to the stretching vibration of C=O in the amide bond -CO-NH- in the aerogel, whereas in the corresponding PI aerogel, the peak disappears here, suggesting the disappearance of the carbonyl group of the amide bond in the precursor after high-temperature treatment [34,35]. Comparing with Figure 4b, which represents the infrared spectra of five different concentrations of aerogels from bottom to top, they show the same vibrational telescoping tendency, and the analysis of the infrared spectra shows that there are two relatively strong emission peaks, they appear in 1777 cm^−1^ and 1717 cm^−1^, which are symmetric vibration of carboxylate C=O corresponding to the amide group as well as asymmetric telescoping vibration, respectively; this peak at 1375 cm^−1^ refers to the stretching vibration of the C-N bond of this CLPI aerogel. Meanwhile, we also observe a characteristic peak at 738 cm^−1^ on the right side, which is ascribed to the bending vibration of the C=O around the acyl ring [35]. These indicate that the PAA aerogel has been completely thermoimidized. Summing up the characteristic peaks of the infrared spectrum, the polyimide aerogels have been completely synthesized.

**Figure 5:**
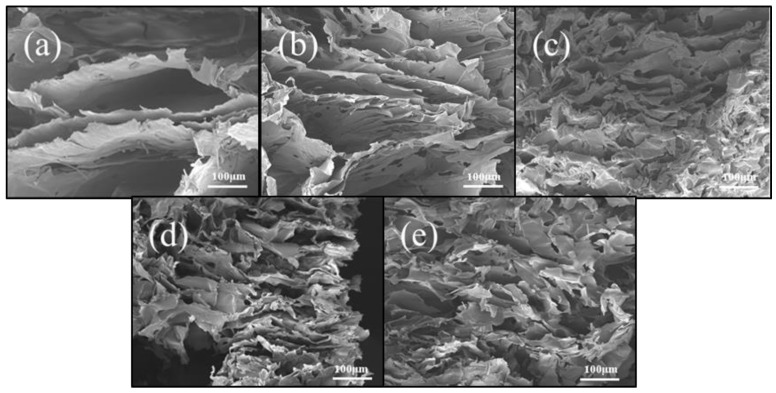

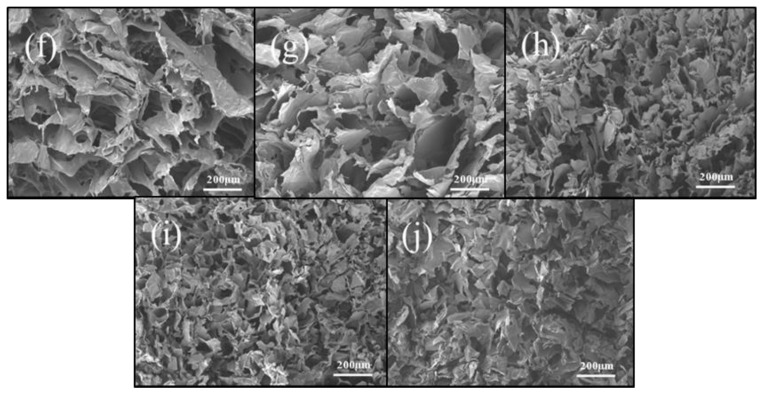
(**a**–**e**) Represent the SEM of the lamellar structures of CLPI aerogels with concentrations of 1–5%, respectively; (**f**–**j**) represent the pore structures SEM of CLPI aerogels with concentrations 1–5%, respectively.

SEM images (Figure 5) were obtained to further understand the micro-structures of CLPI aerogels. The CLPI aerogels as a whole show interconnected and disordered micro-structures. In detail, all of them present laminar and honeycomb pore type structures. As in Figure 5a–e, as the concentration increases, the gap between layers becomes smaller and smaller, and interconnected pore–wall structures are gradually formed between each layer, which become more tightly packed to support the layers, so that denser and denser holes are slowly formed [27,36]. As for the formation of honeycomb shape pore structures, in Figure 5f–j, as the concentration continues to increase, the molecular chains are tightly arranged in the limited space, the structure is denser, and the pore diameter gradually decreases. The formation of these honeycomb and interconnected layer structures can be ascribed to the development of ice crystals in the freezing process and the sublimation of the ice crystals from solid to gaseous. This leads to the development of a pore-layer structure. The higher the concentration, the less air there is inside the aerogel, and the shrinkage during high temperature heating will be noticeably lower than that of the less concentrated samples [37].

However, whether it is a lamellar structure or a honeycomb-type structure, as the concentration increases, the aerogels exhibit increasingly compact structures, and the gradual increase in density under the same volume can confirm the point. At the same time, these compact structures can provide the aerogels with a greater load, and the force will be naturally dispersed to the network structure fibers when the aerogel is compressed or tensile by the outside world, so that the unique structure can give the aerogel excellent mechanical properties [33]. In the heat transfer of aerogel, the ways of calorimetric conduction are solid-phase and gas-phase heat transfer. Moreover, the unique skeletal structure of the CLPI aerogel is filled with air, and their synergistic effect works together to optimize the thermal insulation performance of the aerogel [38,39].

**Figure 6:**
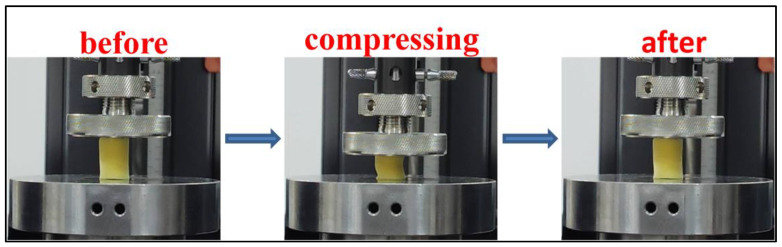
Pre-, mid-, and post-compression strain 50% morphology of CLPI-1 aerogel.

**Figure 7:**
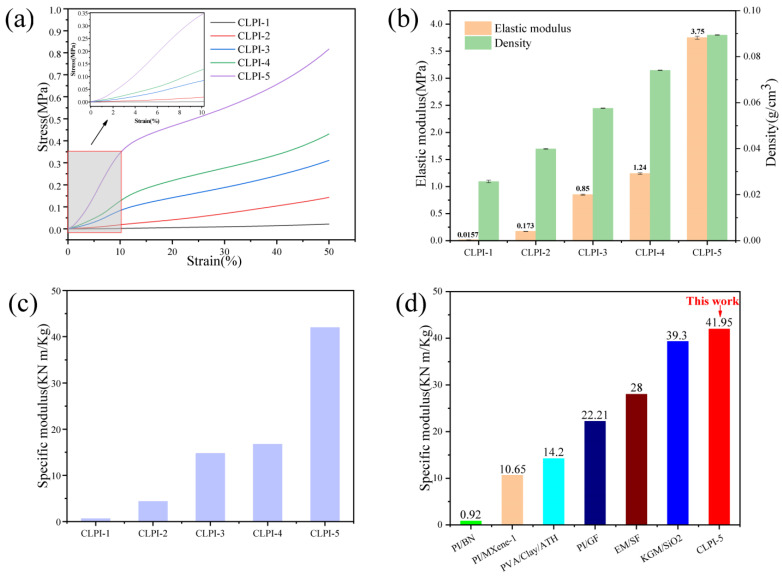
(**a**) Stress–strain curve of aerogels at 50% compressive strain; (**b**) elastic moduli and densities relationship; (**c**) specific moduli of CLPI1-5; (**d**) specific moduli of CLPI aerogel compared to aerogel materials.

We used an electronic universal material testing machine to carry out compression tests on the aerogels. Firstly, each aerogel was condensed with a strain of 50% and compression velocity of 0.12 mm/s to research the effect of different concentrations on the mechanical characteristics of these aerogels. From Figure 6, a comparison of before, during, and after 50% compressive strain of CLPI-1 for example, this aerogel is still able to return to its pervious form after undergoing compression, and has a certain degree of resilience and compressibility. The compressive stress–strain curves obtained were organized in Figure 7a. The elastic moduli are calculated according to the stress–strain curve in the linear growth interval (0–10% strain). The specific modulus is the ratio of elastic modulus to the density of aerogel. The elastic modulus increases with the rising density from 0.016 MPa to 3.75 MPa, as shown in Figure 7b. In Figure 7c, the specific modulus increases from 0.61 KN·m/Kg to 41.95 KN·m/Kg. In addition, the specific modulus of CLPI-5 is compared with that of other aerogels [40] (Figure 7d). CLPI-5 shows a much higher specific modulus compared to that of others like PI/BN nanocomposite aerogel [41], nanosheet PI/Mxene-1 [42], PVA/clay/ATH polymer clay aerogel [43], PI/GF glass fiber composite aerogel [44], EM/SF aerogel [45], and KGM/SiO_2_ composite aerogel gels [46]. As the concentration increases, the mechanical properties of aerogels are also enhanced, leading to smaller pore volume, larger density, and a more compact cross-bonding structure. As a result, when subjected to the external load, the force is dispersed and transmitted in the internal structure, and it will withstand more load under the same strain. Therefore, the overall outstanding mechanical performances are realized [33,38,39,47,48].

**Figure 8:**
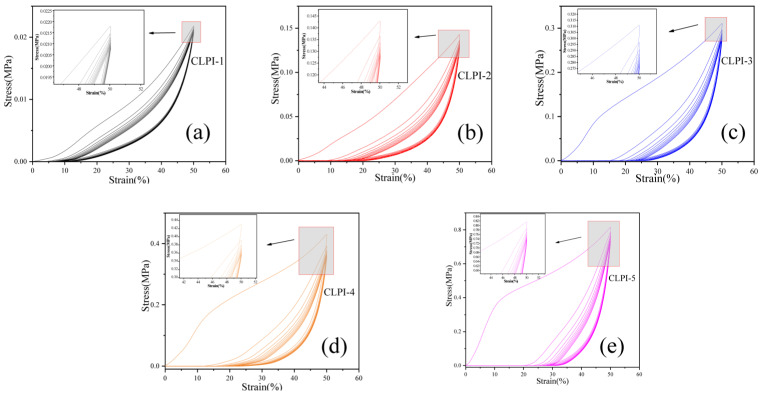
(**a**–**e**) Represent stress–strain curves of CLPI aerogels with concentrations of 1–5% cycling ten times at 50% compressive strain, respectively.

In addition to this, we also performed cyclic compression tests on the aerogels, cycling ten times, with the same compressive strain of 50%, keeping the original compression rate (0.12 mm/s) to study the cyclic compression features of the aerogels. The cyclic compression curves for the five different concentrations are shown in Figure 8. According to the curves, it can be concluded that the aerogel structure was already crushed at that strain for CLPI3-5. CLPI1 and CLPI2 produce compressive stresses of 0.02 MPa and 0.14 MPa during the first compression, respectively. After ten cycles of compression, the compressive stresses of 0.02 MPa and 0.13 MPa were still generated by CLPI1 and CLPI2. The lost energy may be due to the friction between the skeletons during compression and the irreversible damage collapse of some structures, when the air was completely expelled to reach the tight compaction stage [49]. There was an impact on the resilience of the aerogel. However, compared with the first compression, a higher stress can still be maintained, indicating that the aerogel has great mechanical properties and a more stable skeleton structure [33,38,39,47,48]. These reasons are mainly the strong skeleton formed inside the aerogel and the support of the structures by the van der Waals forces generated between the bonds [50,51,52,53].

**Figure 9:**
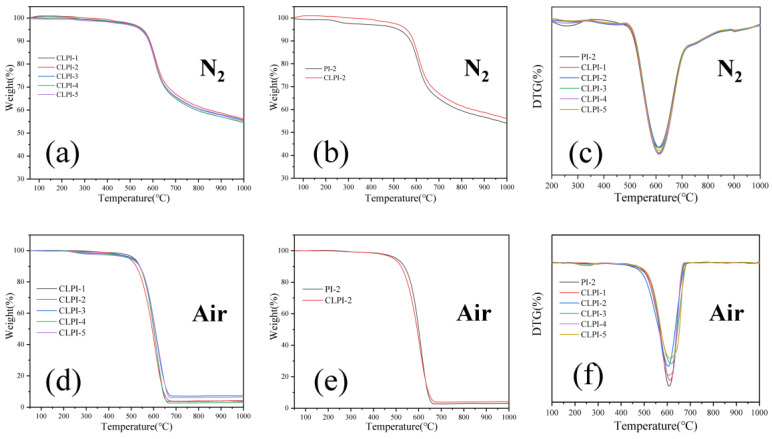
(**a**) TG curves of CLPI1-5 in nitrogen atmosphere; (**b**) TG curves of CLPI-2, PI-2 in nitrogen atmosphere; (**c**) DTG curves of PI-2, CLPI1-5 in nitrogen atmosphere; (**d**) TG curves of CLPI1-5 in air; (**e**) TG curves of PI-2, CLPI-2 in air; (**f**) DTG curves of PI-2, CLPI1-5 in air.

To investigate the thermal stability and thermal insulation quality of aerogels, thermogravimetric analysis was performed and the results are shown in Figure 9a–c. The mass loss of all samples can be divided into two phases in N_2_. The first stage of mass loss (0–5%) could be attributed to the evaporation of water from the samples [49], the damage of the residual organic solvent, DMAc, and the destruction of TEA molecules that may be left on the molecular chain of the PI molecules. At the temperature of 5% mass loss (T_5%_ is around 550 °C), i.e., the second stage, the samples begin to decompose, the aerogel structures are damaged at high temperature, the intermolecular forces are weakened, the chemical groups are broken, and the mass continues to drop [54]. The temperature T_10%_ at 10% mass loss is around 580 °C. Comparing the samples CLPI-2 and PI-2, the initial decomposition temperature (T_5%_) of PI-2 is 519 °C, while the T_5%_ of CLPI-2 is 556 °C; the T_10%_ is 573 °C and 586 °C, respectively, and the mass residuals are still lower than those of CLPI-2 when the maximum temperature is reached. It demonstrates that the cross-linking type is able to reinforce the thermal stability quality to the aerogel effectively. From the DTG of Figure 9c, the thermal decomposition rate of the sample reaches its maximum at about 608 °C (the maximum decomposition temperature T_max_), and finally reaches around 55% of the mass remaining at 1000 °C, which reveals that the samples have a stable thermal nature and high temperature resistance, as they are able to maintain high quality at high temperatures.

Also, the heat loss curves under air atmosphere show the same trend, as shown in Figure 9d–f, with a 5% mass loss also due to evaporation of water from the aerogel and volatilization of organic solvents [49]. T_5%_ and T_max_ are around 510 °C and 610 °C, respectively, the mass is stabilized at around 660 °C, with a residual of only about 5%, which, compared to that under nitrogen atmosphere, is reduced. Because oxygen that exists in the air is more active in nature at high temperatures and participates in the reaction with the aerogel, this results in the production of a carbonized sample. Similarly, as a control, CLPI-2 has a higher temperature of 674 °C and a higher residual than PI-2 when the mass is stabilized, suggesting that the incorporation of cross-linking agents improves the forces of the molecular chains of the aerogel, and then boosts the thermal stability properties [55,56]. In summary, the aerogel has admirable thermal stability characteristic both in nitrogen and air atmosphere. This virtue greatly improves the application scope and effect of its thermal protection.

**Figure 10:**
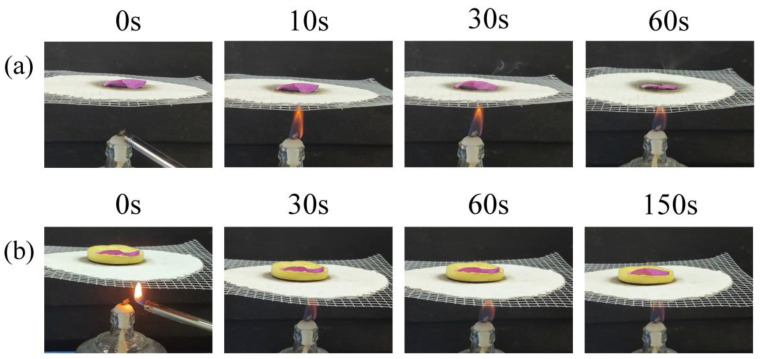
(**a**) Petal without aerogel cushions subjected to heat; (**b**) petal with aerogel cushions subjected to heat.

For the thermal insulating properties of aerogels, we used the comparative heating method, using an alcohol lamp to heat a petal placed on an asbestos mesh and the petal padded with aerogel sheets. Selected at different moments during the heat wilting of the petal, as shown in Figure 10, the petal continued to be heated (Figure 10a), at 30 s withered and wilted, indicating that at high temperature, there had been a case of tissue damage. It continued to be heated, at 60 s, and we can clearly find that the petal blackened, carbonized, and softened. However, when we placed it on the aerogel mat and subjected it to heat (Figure 10b), we observed that the petal did not wither and soften from 0 s to 150 s, and remained brightly colored, because the aerogel mat blocked the heat from the flame for the petal. This demonstrates the remarkable thermal insulation properties of CLPI aerogel.

**Figure 11:**
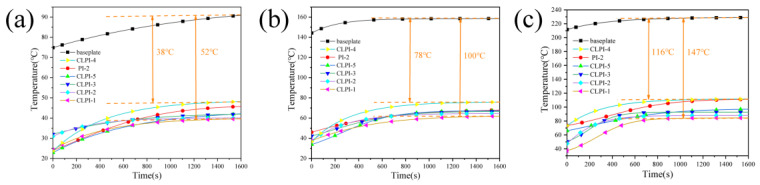
(**a**–**c**) Temperature profiles of heating table, PI-2, and CLPI1-5 at 100 °C, 200 °C, and 300 °C, respectively.

Further, we used a heating table, thermocouples, and temperature sensors to test the insulation performance of the aerogels. These samples of CLPI (1–5) and PI-2 (as a comparison) were taken on the heating table, and heated for 1600 s (when the temperature was stabilized) at 100 °C, 200 °C, and 300 °C. Thermocouples were used to test the thermal insulation performance of the PI and CLPI at the height of 10 mm. The time–temperature curves were obtained (Figure 11). CLPI1 presents the highest thermal insulation performance. On the contrary, the temperature difference is the lowest for the sample CLPI4. With the rise in the concentration, the 3D structure of the aerogels are more compact and the pore sizes are reduced. As a result, the heat mainly transferred through the pore wall structure [57]. The temperature differences for CLPI1 between the heating sink and the aerogels reach a maximum of 52 °C, 100 °C, and 147 °C when the hot part is ~90 °C, ~160 °C, and 230 °C. This result also fully demonstrates that the cross-linked aerogels have great heat insulation performance [58,59,60].

**Figure 12:**
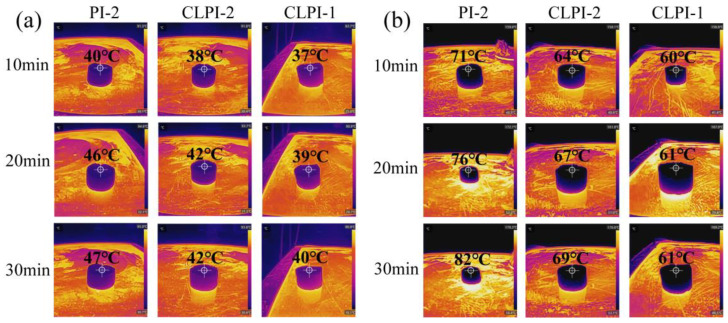
(**a**,**b**) Thermal infrared images of the top of PI-2, CLPI-2, and CLPI-1 at 100 °C and 200 °C, respectively.

To describe the thermal insulation influence more practically, we demonstrated the relevant performance of the aerogels PI-2, CLPI-2, and CLPI-1 by using an infrared thermography camera. We placed these 10 mm aerogels on a heating table and continuously heated at 100 °C and 200 °C for 10 min, 20 min, and 30 min. Then we used a thermal infrared imager to scan the temperature of the top. Figure 12a,b presents the top temperatures of PI-2, CLPI-2, and CLPI-1, which are 47 °C, 43 °C, and 39 °C, respectively when heated continuously for 30 min at 100 °C; when heated continuously for 30 min at 200 °C, the temperatures are 82 °C, 69 °C, and 61 °C. This temperature difference effect proves the splendid heat insulation performance of aerogel and the outstanding role of the cross-linking agent in heat insulation.

**Figure 13:**
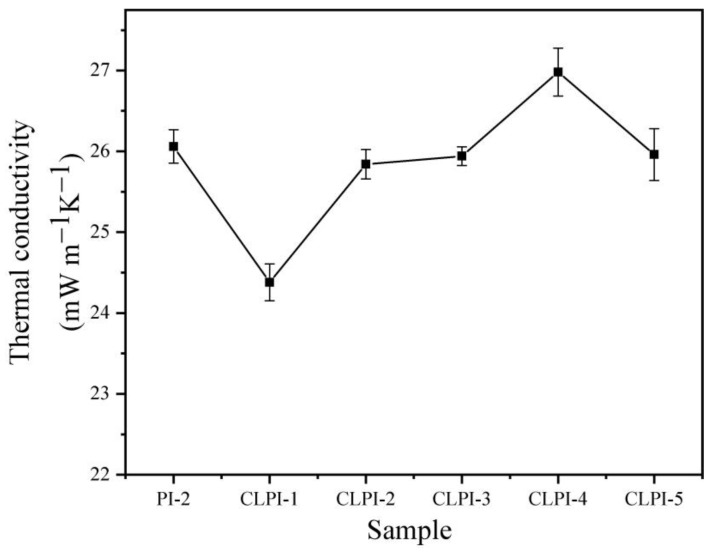
Thermal conductivity of PI-2 and CLPI1-5 at room temperature.

The thermal conductivities of PI and CLPI aerogels at room temperature are expressed in Figure 13. The thermal conductivity of CLPI-1 is as low as 24.4 mW·m^−1^ K^−1^, and as the concentration increased, the coefficients show a tendency of increasing and then decreasing. It is the same trend as that of the thermal insulation curve. This is because as the concentration augments, the density of the aerogel increases, the pore walls are tightly arranged, and the heat transfer is mainly manifested by the synergistic effect of the solid phase λ_s_ of the pore walls and the gas phase transfer of the air λ_g_ [61,62]. As the pore wall of the aerogels become denser, the pore walls act as the main thermal resistance, reducing the heat transfer through the gas. As a result, the thermal conductivity decreases from 26.1 mW·m^−1^ K^−1^ (PI-2) to 25.8 mW·m^−1^ K^−1^ (CLPI-2). This promotion can be attributed to the appending of TAB. It stabilizes the pore–wall thermal resistive interface of the porous layer network structure and reduces the thermal conductivity of the gas phase. Generally speaking, these aerogels exhibit very small thermal conductivities. They can be applied in an extensive scope of thermal protection [57].

**Figure 14:**
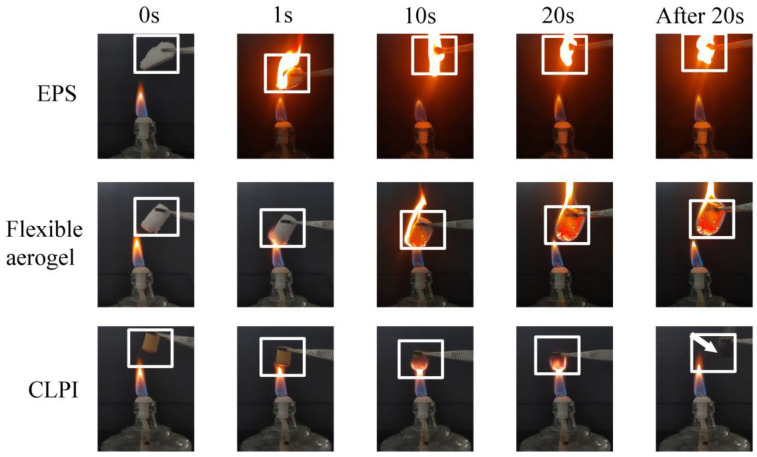
Burning of aerogel and different protective materials at different moments when exposed to fire.

As a new multifunctional material, it needs to not only have excellent performance in thermal insulation, but the flame retardant aspects of the application are also critical. We used cushioning insulation material polystyrene foam, flexible silica aerogel, and polyimide aerogel with an alcohol lamp for combustion. We observed the three in contact with the fire in different periods of time during the combustion situation. From Figure 14, we find that the polystyrene foam was ignited immediately when in contact with the flame, then it continued to burn. After 20 s, the foam was completely burned into black solid particles. The irritating black smoke was produced in the whole process. When flexible silica aerogel was in contact with the flame, the contact surface was ignited, and in 10 s was basically all ignited. Not only that, but the interior was also burning. In 20 s, we moved away from the flame, flexible silica aerogel still continued to burn, as it did not have a tendency to self-extinguish. On the contrary, when the polyimide aerogel was in contact with the flame, the contact surface was carbonized by the flame. The color slowly became black, this phenomenon persisted for 10 s–20 s. At the same time, the carbonization became deeper. We were able to observe that the flame was blocked by the aerogel and dispersed on both sides. However, it was found that there was not a flame burning phenomenon of the aerogel after moving away in 20 s. This indicates that the CLPI aerogel has an excellent flame-retardant performance.

## 3. Conclusions

In this paper, different series of cross-linked polyimide aerogels were prepared by mixing and cross-linking the thermal insulating cross-linking agent 1,3,5-tris(4-aminobenzylamino) benzene (TAB) with polyamic acid solution. That forms a three-positioned network-type structure, by using the freeze-drying and thermimide methods and controlling the concentration of an aqueous polyamic acid solution. The appending of TAB as a “connecting” effect to the structure inhibits the volume contraction of the aerogel to a certain extent (from 36% in PI-2 to 33% in CLPI-2). The TAB also improves the thermal insulation and heat resistance of the aerogel (T_5_% in nitrogen from 519 °C in PI-2 to 556 °C in CLPI-2). Moreover, as the concentration of the aqueous polyamides improves, the shrinkage decreases (from 40% to 28%), the structure becomes denser and the modulus of elasticity increases dramatically, from 0.0157 MPa to 3.75 MPa. For its thermal insulation and thermal stability properties, the initial decomposition temperature T_5_% reaches 556 °C. The synergistic heat conduction between the gas and solid phases results in an aerogel thermal conductivity of 24.4 mWm^−1^ K^−1^. The excellent results are attributed to the unique three-dimensional networked pore-layer structures of the aerogel as well as the stabilizing effect of TAB on the structures of the porous layer. All of them ensure it has a great prospect for the field of thermal insulation and protection.

## 4. Materials and Methods 

### 4.1. Materials

All of 4,4-Diaminodiphenyl ether (ODA, 98%), 3,3,4,4-Biphenyltetracarboxylic acid dianhydride (BPDA, 97%), N,N-dimethylacetamide (DMAc, 99%), and 1,3,5-Tris(4-aminobenzylamino)benzene (TAB, 97%) were purchased from Aladdin Bio-Chem Technology Co., Ltd. (Shanghai, China). Triethylamine (TEA) was purchased from Sinopharm Chemical Reagent Co., Ltd. (Shanghai, China).

### 4.2. Preparation of Cross-Linked Water-Soluble Polyamides

A total of 8.41 g ODA was dissolved in 140 mL DMAC in a flask with mechanical stirring for 40 min until ODA was completely dissolved. Then, 12.48 g BPDA was added, placing the flask into ice water. The solution was continuously stirred for ~4 h to prepare a yellow, transparent, and viscous polyamic acid solution with a concentration of 10%. Then, 0.49 g TAB was added and stirred for 1 h to cross-link. The resulting yellow solution was slowly dumped into water. Then the white filamentous sediment appeared and was washed with water until it became clarified. Finally, the clarified sediment was dried at 60 °C about 12 h. Then, the yellow powdered polyamic acid salt (PAAs) was obtained.

### 4.3. Preparation of Aerogel Precursors

Since polyamides are soluble in alkaline solutions, we injected TEA (0.5 g) into deionized water (49.5 g) to form a TEA alkaline aqueous solution (Ph = 11–12), and a PAA solution at a concentration of 1% was prepared by dissolving 0.5 g of PAAs in TEA aqueous solution with stirring and, similarly, PAA solutions at concentrations of 2%, 3%, 4%, and 5% were also prepared. Then, this was poured into cylindrical molds in batches and frozen in a freezer (−60 °C) for about 12 h. This was transferred to the lyophilizer for freeze drying for 48–72 h to obtain white polyimide aerogel precursors.

### 4.4. Preparation of Cross-Linking PI Aerogel

We placed the obtained aerogels precursor samples in a drying oven with a gradient temperature rise for thermal imidization, dried sequentially at 80 °C,100 °C, 120 °C, 150 °C for 30 min, with a temperature rise rate of 4 °C/min at each stage. Then, they were dried sequentially at 180 °C, 210 °C, 230 °C for 60 min, with a temperature rise rate of 2 °C/min, ultimately obtaining yellow solid polyimide aerogels. The cross-linked aerogels prepared with different concentrations were named CLPI-1, CLPI-2, CLPI-3, CLPI-4, and CLPI-5, and a pure aerogel named PI-2 with 2% concentration without cross-linking agent was prepared as a comparison.

### 4.5. Characterization

The bulk density of aerogel was obtained by measuring its volume and mass with vernier calipers and pallet balance, and we used the following formula method to calculate shrinkage percentage of the aerogel:

φ = (1 − V/V_0_) × 100% (1)

where volume (V_0_) represents the polyimide aerogel precursor, and volume (V) refers to the polyimide aerogel.

Polyimide aerogels were subjected to FTIR using an intelligent Fourier-transform infrared spectrometer (Nexus) (Thermo Fisher Scientific, America) to analyze and characterize the structures. Microstructure of the samples was analyzed using a field-emission scanning electron microscope (JSM-IT800) (Nippon Electronics Corporation, Japan). Using an electronic universal material testing machine (Instron 5967) (Instron, America) the samples were compressed at 50% compression strain and cycled at 50% compression strain for ten cycles at a compression rate of 0.12 mm/s to measure the mechanical characteristics. To explore the thermal stability performances of the aerogels, the comprehensive thermal analyzer (STA449F3) (Netzsch German) was applied, the samples were heated with a rate of 10 °C/min from room temperature to 1000 °C. Flame-retardant properties of aerogels was studied by the vertical burning tester.

## Data Availability

All data and materials are available on request from the corresponding author. The data are not publicly available due to ongoing research using part of the data.
